# Level of asthma control and its determinants among adults living with asthma attending selected public hospitals in northwestern, Ethiopia: using an ordinal logistic regression model

**DOI:** 10.1186/s40733-022-00087-3

**Published:** 2022-08-27

**Authors:** Eyayaw Ashete Belachew, Sumeya Tadess, Mekuriaw Alemayehu, Emneteab Mesfin Ayele

**Affiliations:** 1 Department of Clinical Pharmacy, University of Gondar, P. O. Box – 196, Gondar, Ethiopia; 2 Institute of Public Health, University of Gondar, P. O. Box - 196, Gondar, Ethiopia

**Keywords:** Asthma, Asthma control, Determinant, Ethiopia

## Abstract

**Background:**

Asthma is a major public health challenge and is characterized by recurrent attacks of breathlessness and wheezing that vary in severity and frequency from person to person. Asthma control is an important measure of health outcomes of the patients with asthma and reflecting the impact of an illness and its treatment from the patient’s perspective. Therefore, this study assessed the asthma control levels and their determinants among adults living with asthma in selected public referral hospitals in northwestern Ethiopia.

**Materials and method:**

A multicenter institutional-based cross-sectional study was conducted in North-western Ethiopia, from October to December 2021. A systematic random sampling technique was employed to recruit the study participants. Bi-variable and multivariable ordinal logistic regression was used to determine the independent predictors of asthma control levels. A *p*-value of < 0.05 was considered as statistically significant.

**Result:**

A total of 409 patients were included in the final analysis. Asthma was controlled by 28.9% with 95%CI (24.7, 33.5) people who have asthma. Regarding the potential predictor of asthma control level, being male (AOR = 6.5, 95%CI (1.28, 32.44), Married (AOR = 3.62, 95%CI (1.28, 10.27), healthcare provider adherence to guideline usage (AOR = 8.4,95%CI (2.7, 26) and non-fuel users (AOR = 6.0, 95%CI (1.5, 22.5) were variables that increase asthma control. However, non-adherent to medication (AOR = 0.16, 95%CI (0.059, 0.48), low level of patient enablement (AOR = 0.19, (95%CI) (0.08, 0.49) and poor relationship with healthcare provider (AOR = 0.024,95%CI (0.02, 0.23) were variables that significantly decreased asthma control level.

**Conclusion:**

The findings indicated that asthma control remains suboptimal in a large proportion of patients with asthma in the study setting. Socio-demographic, clinical, healthcare-related, and medication-related variables were significantly associated with asthma control. Therefore, our study highlights multifaceted interventions, including comprehensive asthma education along with an integrated treatment plan to improve asthma control and quality of life.

## Background

Asthma is a heterogeneous disease, usually characterized a chronic airway inflammation and defined by the history of respiratory symptoms such as wheezing, shortness of breath, chest tightness, and cough that vary over time and in intensity, with variable expiratory airflow limitation [1, 2]. Environmental pollution, upper respiratory tract infection, household pests, colds, laughter, tobacco smoke, and bad smell were the common triggers of asthma [3]. Seasonal exposure to allergens is also a risk of asthma triggers such as pollen, and mold. Pets, cockroaches, and dust mites [4–7]. Dust mites were the commonest asthma trigger allergens identified in Ethiopia, particularly in the study area [5].

Asthma has rated a serious public health problem and its prevalence and burden kept increasing globally in all age groups [8]. Globally, it is a big concern and the most important disorder in terms of the extent and duration of disability [1, 9]. In Africa, the prevalence in the total population increased from 74.4 million to 119.3 million within just two decades (1990–2010) [10]. Reports from Sub-Saharan- Africa countries also showed a surge in prevalence; whereas the prevalence in Ethiopia showed that ranged from 10.2–13% [11] of the total population. The prevalence of uncontrolled disease in Ethiopia was 53.3%. However, a recent study in Aksum revealed that the prevalence of uncontrolled asthma is 71.67% [12]. The level of asthma control has a significant impact on Disability Adjusted Life Years [13], increases financial burdens [14], and will has an effect on daily activities and causes physical, emotional, and social limitations [15].

Studies have revealed that asthma is under-diagnosed and undertreated, especially in low-income countries and rural settings are the most affected countries like Ethiopia [10]. Despite greater progress over the past 30 years in terms of pathophysiology and management of asthma, studies have shown that this disorder remains largely uncontrolled [16]. Moreover, regardless of the widespread accessibility of therapies reported as highly effective in randomized controlled trials, variable levels of asthma control have been shown in several real-life studies using well-validated self-assessment questionnaires [17, 18]. Several factors predict the level of asthma control, for instance, age, gender, smoking, comorbidities, seasonal worsening, exposure to allergens/irritants/ triggers, and treatment-related issues such as disease perception, adherence to medication, and belief about medication [19–22].

Some studies have been conducted at the level of asthma control and associated factors in Ethiopia. But these studies are conducted on single centered and are unable to capture the ordinal nature of the level of asthma control since treatment optimization of patients with asthma is based on the level of asthma control. Therefore, we applied the multilevel ordinal logistic regression model to get a reliable estimate and avoid the loss of information. This study has both public health and methodological significance. Concerning the public health perspective, this study is the first of its kind to assess the level of asthma control and its determinants in the study area. As there are no studies published in northwestern Ethiopia and Amhara regional state as a whole, this study will be an important input to assist an existing effort to improve asthma control and target the associated factors. Additionally, the finding will provide good evidence for in the study setting to planning interventional strategies, a body of knowledge for further studies that might be conducted on related topics or for organizations working with asthma patients and might have important clues to characterize and stratify patients at follow-up care and optimize care based pertinent precipitants. Regarding the methodological perspective, as you can see from the previously published literature, asthma treatment was based on the binary outcome by categorizing controlled/uncontrolled but as you can understand, treating poorly and partially controlled asthma as uncontrolled is not statistically appropriate since there is a loss of information because the factor responsible for partial control asthma may not be similar to the factor that can cause poorly controlled asthma. As a result, this study assessed the asthma control levels and their determinants among adults living with asthma in selected public hospitals in northwestern Ethiopia.

## Methods and materials

### Study setting and design

A multi-centered institutional based cross-sectional survey through patient interviews and medical record review was conducted from October to December 2021 at three public comprehensive specialized hospitals; University of Gondar Comprehensive Specialized Hospital (UOGCSH), Felege Hiwot Comprehensive Specialized Hospital (FHCSH) and Tibebe Ghion Comprehensive Specialized Hospital (TGCSH) ambulatory care. The University of Gondar Comprehensive Specialized Hospital is a public comprehensive referral health facility present in northern Ethiopia, which serves as a teaching hospital for the University of Gondar College of medicine and health science students. The hospital is located around 738 km far from the capital city of Ethiopia. Asthma follows up runs every on Monday and reviews at least 280–300 patients with asthma monthly as per the UOGCSH records. Felege Hiwot Comprehensive Specialized Hospital is located in Bahir Dar city, which is located in the northwest and 565 km away from the capital of Ethiopia. It is one of the three governmental Hospitals in the city. It has 200 beds and three medical OPD serve for medical patients of which one serves as a referral and follow-up clinics for patients with chronic diseases. The chest clinic of the hospital serves chronic asthma and patients with COPD. Asthma follow-up runs from on Monday to Friday on average reviews at least 190–200 patients monthly as FHRH medical records. Tibebe Ghion Comprehensive Specialized Hospital (TGCSH) ambulatory care is a teaching hospital under the college of medicine and health sciences of Bahir Dar University located in Bahir Dar, Ethiopia. This is one of the 43 governmental hospitals in the Amhara region. The hospital serves more than five million people in a catchment area. This teaching hospital has more than 500 beds and 2000 patients per day in both inpatient and outpatient services. From the OPD service asthma, follow-up runs every Wednesday and reviews on average 70–80 patients per month as FGSH medical records.

### Study population and sampling

Patients with asthma aged 18 years and above who attended the selected hospital’s ambulatory care for follow-up were eligible for this study. Also, the study subjects should have received ICS therapy for the last 3 months to be included. Whereas, patients who were unable to communicate, critically ill, admitted to inpatient departments and uncompleted medical records were excluded. The sample size was determined using a single population proportion formula and 422 individuals included by expecting the proportion of patients with controlled asthma were used from a previous study in Ethiopia which was reported to be 46.7% [14]. 5% absolute precision or margin of error, 5% significance, and 95% confidence level were employed; and 10% contingency was for non-response used. A systematic random sampling technique was used to recruit the study participants. Then, we proportional allocated the study participants proportionally in each selected hospital. Here 224, 142, and 56 patients participated in UOGCSH, FHCSH, and TGCSH, respectively. Respondents were allocated proportionally as per the number of patient flow into the respected hospitals, which were selected through a systematic random sampling method. Finally, the sample was collected within 3 months, this makes the sampling fraction (k-interval) 1695/422 = 4 approximately the initial study subject was selected by the lottery method, and then corresponding medical records were collected, study individuals were chosen by every four person and relevant data were taken. For medical records of the study subjects who met the inclusion criteria were considered and whenever one medical record on hand was ineligible, the next immediate one was selected, and the same approach was followed throughout the data collection procedure.

### Data collection tools and procedures

After reviewing various related literature, the data collection tool was primarily prepared in English. It was then translated into the local language (Amharic) and then translated back to English to ensure consistency. The instrument consisted of five sections; (I) the first section included socio-demographic characteristics of the study participants, (II) the second section included the clinical characteristics, and measurement of asthma control, and (III) the third section contained the instrument to measure ICS treatment adherence (MARS-A), (IV) fourth section contained medication recorded and last (V) consisted of healthcare provider factors. Data collectors had received training on the aims of the study, data collection instruments and producers, and ethical issues. Relevant data were obtained by interviewing participants and reviewing the medical records volunteer study participants. However, if one of the available medical records was ineligible, the next one was considered, and the same approach was followed throughout the data collection. The Asthma Control Test (ACT) was employed in this study to measure the levels of asthma control. The tool is standardized and applicable across populations worldwide [13]. For this study participants, the data collectors administered the questionnaires to participants in a questionnaire-guided interview, mitigating against such obstacles as language barriers and low literacy levels. The ACT tool is a simple test for patients with asthma aged 12 years and above and measures the level of asthma control. It contains 5 questions on a 5-point scale depicting the frequency of asthma symptoms and usage of rescue medication by participants in the last 4 weeks. The overall score was in the range of 5 (worse control) to 25 (total control) [13].

The Medication Adherence Rate Scale (MARS-A) was used to measure the adherence of the patient to their medication [23], and an old version of the Beliefs about Medicine Questionnaire (BMQ) [24] was used to measure the beliefs of the patient in their medication and other different studies [3, 14] were used to develop other related questions.

The belief about Medicine Questionnaire is a 10-item questionnaire that assesses the patient’s beliefs about their prescribed medication. It is composed of two-five item scales that are the specific necessity and specific-concern scales. The specific-necessity scale assesses patients’ beliefs about the prescribed medication to maintain their health now and in the future. However, the scale of the specific concern assesses patients’ perceptions about the adverse consequences of taking medicines related to long-term effects and dependence [25]. The patients’ level of beliefs about medicine (s) before and clinical appointments toward their asthma management care were computed using a 5-point Likert-type scale ranging from strongly disagree = 1 to strongly agree = 5 [26].

Adherence to ICS was measured using the MARS-A, a self-reported adherence tool that demonstrated good test-retest reliability (r = 0.65, *p*-value < 0.001), internal consistency reliability of 0.85, and the sensitivity of 0.82, and specificity of 0.69. The responses were measured using a 5-point Likert scale (1 = Never, 2 = Rarely, 3 = Sometimes, 4 = Often, 5 = Always). Self-reported adherence is reported as the average score of the 10 items (1–5), where higher scores indicate higher levels of reported adherence. High self-reported adherence was defined as a MARS-A score of 4.5 or higher [27].

The modified patient enablement index (mPEI) was used to evaluate the enablement of the patient and a score of more than 6 indicated clinically meaningful enablement. It contains six items: assess the patient’s ability to deal with life, understand the illness, cope with it, keep healthy, remain confident about health, and help oneself [28]. The comorbidity index was done using the Charlson comorbidity index to determine the burden of comorbidity on asthma control and health-related quality of life (HRQOL) [29].

All the above tools were translated into Amharic using translators who were speakers of English and Amharic. The translation was verified for compatibility with the original version by a process of forward and backward translation, performed by persons who were native speakers of Amharic and fluent in English. Face validity of the survey was assessed among three clinical pharmacy teachers for clarity of the questions. Then, the survey was pretested for content, design, readability, and comprehension on 42 people, and modifications were made as necessary so that the survey was simple to understand and answer, yet provided accurate data.

### Data quality management

The data collection instruments were prepared after reviewing similar literature and amendment was performed by considering local clinical settings. The face validity of the questionnaire was checked by three language experts for clarity of questions. Subsequently, the survey was pretested for content, design, readability, and comprehensibility in 10% of study participants who were excluded from the final analysis. Socio-cultural adaptation was performed using recommendations from WHO, and changes were made based on responses. Thus, the survey was easy to understand and respond to while still providing accurate data. The Cronbach alpha was used for tools and medication belief (α = 0.726), MARS-A (α = 0.90), mini-AQLQ (α = 0.898), ACT (0.83), and the role of patient enablement (0.92).

### Operational definition

Asthma control: implies the extent to which the various manifestations of asthma are reduced or removed by treatment.

Well-controlled: It represents asthma control in which a respondent’s score of 20–25 for ACT-like daytime symptoms, night time symptoms, limitations in activities, and rescue medications used to be none in the previous 4 weeks [30].

Partially controlled This stands for the respondent’s score is 15–19 out of 25 according to the ACT tool [30].

Uncontrolled: This stands for the respondent’s score being less than 15 of 25 ACT scores [30].

Highly Adherent to medication: A patient who scored ≥4.5 for the MARS-A was adherent to the controller medication [27].

Low adherent to medication: A patient who scored < 4.5 for the MARS-A was non-adherent to the controller medication [27].

The role of patient enablement means the measure of patient’s ability to understand and cope with life and illness after a consultation with a general practitioner [31].

Patient enablement: There was a minimum score of 0 and a maximum score of 12. A PEI score of ≥6 has been reported as indicating clinically meaningful “enablement” [28].

Optimal: The most favorable interventions and their amounts for achieving asthma control.

Adherence/compliance: are synonymously used. Adherence to guidelines is the degree to which HCP behavior corresponds with the collaborative agreed recommendations from GINA, 2018 guideline. Compliance refers to the extent to which a patient’s behavior matches guideline advice.

### Data analysis

The data was checked for its completeness, and cleanness, then coded and entered into the Epi Information Version 7 database and exported to SPSS Version 26 for analysis. Descriptive statistics, means, median, proportions, tables, and figures, were used to describe the characteristics of the study patients and displayed the study results. First, all the assumptions of the ordinal model were checked (outliers, multicollinearity, and proportional odds) the statistical methods for all variables were tested and those fulfilling the assumptions were entered into further analysis. Additionally, a histogram or normal probability plot of the residuals was used to examine the data distribution and the test indicated that the residuals were approximately normally distributed. Finally, variables with a *p*-value ≤0.2 in bi-variable were entered into a multivariable ordinal logistic regression to determine the independent predictor variables of asthma control. To identify factors associated with asthma control, multivariable ordinal regression was tested and an adjusted odds ratio (AOR) and 95% confidence interval [27] were used to assess the strength and direction of associations between the dependent and independent variables. The statistical significance was declared using a *p*-value less than 0.05. The adequacy of the models was checked by the like hood Chi square-test of the goodness of fit.

### Ethical considerations and confidentiality

The study protocol was approved by a research review committee of the clinical pharmacy department, the University of Gondar with the reference number 132/2021. An official letter of cooperation was written to the chronic ambulatory department of each hospital. The names and addresses of the patients were not documented during the data collection to ensure confidentiality. The information will not be disclosed to anyone in any way and will only be used for the study purpose.

## Result

### Socio-demographic characteristics of the study population

A total of 409 participants were included in the survey with a response rate of 96.7%. The mean (±SD) age of the study participants was 49.82 (±16.1) years and most of them were in the range of 35–64 years. More than half (60.1%) participants were females. More than two-thirds (71.9%) respondents were married and about three-fourths (73.3%) were urban dwellers. The biomass fuel users were represented (88.5%) and from these Charcoal and wood were used as the main sources of food preparations (82.4%). The majority (87.5%) of study subjects had no smoking history (Table 1).

**Table 1:** Socio-demographic characteristics of adult patients with asthma attending ambulatory care units of selected public hospitals in Northwestern Ethiopia, 2021 (*N* = 409)

Variable	Number (%)
Age, mean (±SD)	49.82 (±16.1)
18–34	83 (20.3%)
35–64	238 (58.2%)
≥65	88 (21.5%)
Gender	
Male	163 (39.9%)
Female	246 (60.1%)
Residency	
Urban	300 (73.3%)
Rural	109 (26.7%)
Marital Status	
Single	41 (10%)
Married	294 (71.9%)
Divorced	16 (3.0%)
Window	58 (14.2%)
Education level	
Unable to read or write	174 (42.6%)
Primary education	72 (17.6%)
Secondary education	89 (21.8%)
Higher institute	74 (18.1%)
Occupation	
Government employee	115 (28.1%)
Farmer	66 (16.1%)
Homemaker	112 (27.4%)
Merchant	54 (13.2%)
Student	62 (15.1%)
Yearly Income level (±SD)/ birr	414,338.26 ± 42,641.0
Access of healthcare	
Insurance	173 (42.3%)
Free	83 (20.3%)
Out of pocket	153 (37.4%)
Biomass use	
Yes	362 (88.5%)
No	47 (11.5%)
Fuel type	
Kerosene	15 (3.7%)
Charcoal and wood	337(82.4%)
Ethanol	6 (1.5%)
Disel-fuel	13 (3.2%)
Smoking	
Never smoker	358 (87.5%)
Current smoker	13 (3.2%)
Ex-smoker	38 (9.3%)

### Clinical characteristics and the triggering factors of asthma exacerbations

**Fig. 1:**
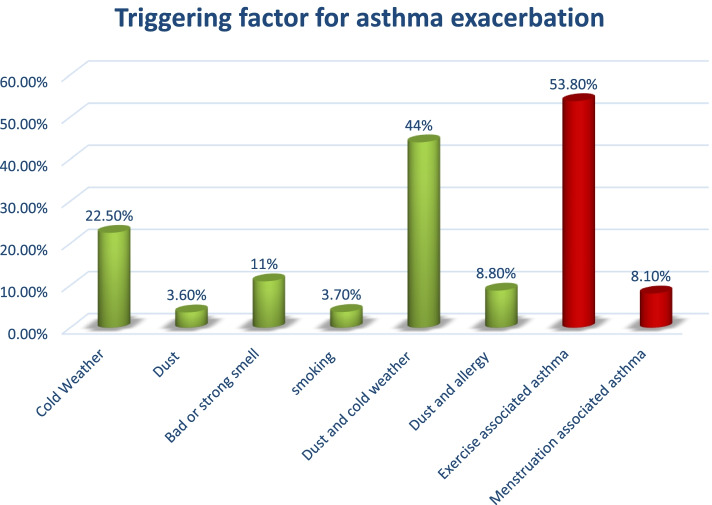
Triggering factors for asthma exacerbations. Note: one participant may choose more than one so, the percentage maybe be greater than 100%

More than three-fourths (76%) of the sampled study were diagnosed with asthma after 12 years of age. The median (IQR) duration of the medications after the onset of the condition was 3(6) years. In the last one-year period, more than half (53.1%) had at least one episode of asthma exacerbation and 95.4% of them had at least one triggering factor for their exacerbations. The dust particles combined with cold weather were the leading cause of the exacerbations, followed by cold weather alone (44.5%) and 22.5%, respectively. More than half (53.5%) of them had exacerbated their symptoms during exercise. Regarding the female respondents, only (8.1%) had reported menstruation-induced asthma exacerbations (Fig. 1). After maintenance therapy was initiated, the median (IQR) time to visiting an emergency department was 11.95 (13.6) months. Most patients had taken the medication for 1–5 years. Regarding the effect of class and type of concurrent medication in asthma control, there were no significant impacts shown in our study. Almost one-third (33.3%) of the patients were hospitalized in the last 12 months and 6.1% were sent to the ICU unit. Concerning the medication experiences, more than one-third (37.2%) used oral steroids. According to GINA-based severity classifications, (56.7%) respondents had moderately persistent and (26.2%) had mild persistent asthma (Table 2).

**Table 2:** Clinical characteristics and triggering factors of adult patients with asthma attending ambulatory care units of selected public hospitals Northwestern Ethiopia, 2021 (*N* = 409)

Variable	Number (%)
Age of onset (year)	
< 12 years	98 (24%)
≥ 12 years	311 (76%)
Duration on medication (years median (IQR))	3 (6)
< 1 year	93 (22.7%)
1–5 years	180 (44%)
5–10 years	87 (21.3%
> 10 years	49 (12%)
Exacerbations in the last 12 months	
Yes	217 (53.1%)
No	192 (46.9%)
Time to emergency visit after start of maintenance therapy (In month) (IQR)	11.95 (13.6)
Hospitalization in the last 12 months:	
Yes	136 (33.3%)
No	273 (66.7%)
Admitted to ICU (intubated) in the last 12 months	
Yes	25 (6.1%)
No	284 (93.9%)
Oral steroid use	
Yes	152 (37.2%)
No	257 (62.8%)
Oral SABA use	
Yes	253 (61.9%)
No	156 (38.1%)
Asthma severity stage	
Intermittent	25 (6.1%)
Mild persistent	107 (26.2%)
Moderate persistent	232 (56.7%)
Severely persistent	45 (11%)
Lung function test (±SD)	
FEVs 1, percent predicted	74.3 (5.31)
FVC 1, percent predicted	78.08 (5.63)
FEV1,% predicted	82.3 (9.45)

### Patterns of antiasthmatic drug drugs

Drug usage patterns suggested that multiple-drug therapy (two or three) drug combinations were used by almost all patients (99%). More than three-fourths (76.5%) had Salbutamol puff PRN and Beclomethasone puff Bid, followed by Salbutamol puff PRN and Beclomethasone puff Bid and Prednisolone (11.5%). Oral and inhaled short-acting beta agonist (SABA) medications were the two most commonly used combinations of medicines used by patients with uncontrolled asthma (Table 3). The Medication Adherence Rating Scale showed that (13.9%) of patients had a high level of adherence to the prescribed controller medication (s). A large proportion of 305 (74.6%) respondents were on the optimal dosage of asthma medication at various steps. Asthma medication combination therapy was appropriately selected for (71.1%) subjects. In the prescribing pattern, about 21.8% of prescribers had not adhered to current guideline recommendations. Nearly three-fourths (74.3%) of the respondents, were adequately educated about people who have asthma and its management. Of the participants, 91.7 and 89.5% had a good relationship with healthcare providers and were satisfied with the care, respectively (Table 3).

**Table 3:** Drug usage pattern among adult patients with asthma and anti-asthmatic medication adherence at ambulatory care units of selected public hospitals Northwestern Ethiopia, 2021 (*N* = 409)

Variable	Number (%)
Salbutamol puff PRN + Beclomethasone puff Bid	313 (76.5%)
Salbutamol puff PRN + Prednisolone oral daily	25 (6.1%)
Salbutamol puff PRN + Beclomethasone puff Bid + Prednisolone	47 (11.5%)
Salbutamol puff PRN + Beclomethasone puff Bid+ theophylline daily	6 (1.5%)
Salbutamol puff PRN + Budesonide puff bid	4 (1%)
Theophylline +Salbutamol puff PRN	4 (1%)
Theophylline +Salbutamol puff PRN + prednisolone daily	1 (.2%)
Fluticasone puff +Salbutamol puff PRN	2 (0.5%)
Beclomethasone puff bid+ salbutamol puff PRN + symicort	2 (.5%)
Alimetamin	3 (.7%)
Oral SABA +salbutamol puff	2 (0.5%)
Medication adherent	
High	57 (13.9%)
Low	352 (86.1%)
Dose of the anti-asthmatic drug	
Optimal	305 (74.6%)
Suboptimal	104 (25.4%)
Appropriateness of drug selection based on severity	
Appropriate	291 (71.1%)
Inappropriate	118 (28.8%)
Healthcare providers adherence to guidelines	
Yes	320 (78.2%)
No	89 (21.8%)
Patient information provided	
Yes	304 (74.3%)
No	105 (25.7%)
Patient relationship with healthcare provider	
Good	375 (91.7%)
Poor	34 (8.3%)

### Comorbidities and concurrent medications

**Fig. 2:**
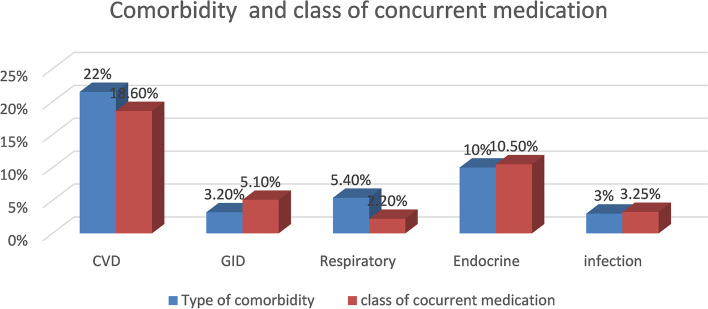
Type of comorbidity and commonly used class of concurrent medication among adult asthmatic patients. CVD: cardiovascular GI: gastrointestinal Diseases

Regarding the Charlson comorbidity index (CCI), almost 60 % of the respondents were found in the mild range of categorizations and about 40 % of the individuals had comorbidities. More than one-third (38.3%) patients were prescribed concurrent medications (Table 4). This shows that a large number of comorbidities were documented in this study. A large proportion of participants had (21.5%) cardiovascular disease followed by diabetes mellitus (10%) and the two most commonly used classes of concurrent medication were cardiovascular drugs 18.6% followed by endocrine drugs 10.5% (Fig. 2).

**Table 4:** Charleson comorbidity index, comorbidity history and concurrent medication use among asthmatic patients

Variables	Number (%)
Charleson comorbidity index	
Mild	241 (58.9%)
Moderate	132 (32.3%)
Sever	36 (8.8%)
Comorbidities	
Yes	162 (39.6%)
No	244 (59.4%)
Use of concurrent medication	
Yes	157 (38.4%)
No	252 (61.6%)

### Asthma control tests and determinants of asthma control

The mean score on the asthma control test was 16.46 (±4.1). Out of the 409 participants, 118 (28.9%), 95%CI (24.7, 33.5) had well-controlled asthma, 143 (35%) had partially controlled and 148 (36.2%) had poorly controlled asthma. Ordinal logistic regression was used to identify the independent predictors of asthma control.

In the multivariable ordinal logistic regression, several variables were substantially associated with asthma control levels. As to the regression analysis, the odds of asthma control among male participants were about 6.5 times higher than females, AOR of 6.5 (95% CI (1.28, 32.44), *P* = 0.024). Compared with the widowed respondents, married patients had increased their asthma control levels by 3.6 times (AOR = 3.6, 95% CI (1.28, 10.27), *P =* 0.0015). Biomass non-users for cooking had about 6 times higher odds of asthma well-control status than the users (AOR = 6.04, 95% CI (1.59, 22.87), *P* = 0.008). Similarly, the HCP who adhered to the treatment guidelines had 7.4 times higher odds of a well-controlled asthma level than those who had not adhere the guidelines (AOR = 8.4, 95%CI (2.7, 26), *P* < 0.001). The odds of the well-controlled asthma status in patients with low adherence to ICS were decreased by 83% compared with those who had high adherence to their medications (AOR = 0.16, 95%CI (0.059, 0.481), *P* < 0.001). Also, low level of patient enablement and poor relationship with the healthcare provider decreased the probability of being well-controlled asthma by 80.7% (AOR = 0.19, 95% (0.08, 0.499), *P* = 0.001) and 97.6% (AOR = 0.024 (0.002, 0.23), *P* = 0.001), respectively. One of the interesting aspects identified in our study is a direct relationship between the level of asthma control and the lung function test (Table 5).

**Table 5:** Independent predictors of asthma control among asthmatic patients at Northwestern, Ethiopia, bi-variable analysis and multivariate analysis (*N* = 409)

Variable	COR (95% CI)	*P*-value	AOR (95% CI)	*P*-value
Age	0.98 (0.97, 0.99)	**0.012**	0.99 (0.96, 1.02)	0.52
CCI score	0.86 (0.76, 0.98)	0.034*	0.72(0.51, 0.96)	0.37
Medication burden in number	0.78 (0.65, 0.94)	0.012*	0.80 (0.47, 1.35)	0.41
Belief in medication means score	0.62 (0.44, 0.89)	0.010*	0.59 (0.30, 1.16)	0.13
Lung function test				
FEVs 1, percent predicted	1.10 (1.06,1.14)	**< 0.001**	1.05 (1.00,1.10)	0.023
FVC 1, percent predicted	1.12 (1.08,1.07)	**< 0.001**	1.08 (1.04,1.13)	< 0.001
Gender				
Male	1.59 (1.10, 2.30)	0.013*	6.45(1.28,32.44)	0.024*
Female	1		1	
Marital status				
Single	3.54 (1.65, 7.61)	0.001*	0.04 (0.01, 1.28)	0.075
Married	1.82 (1.06,3.09)	0.028*	3.63(1.28,10.28)	0.015*
Divorced	1.43 (0.54,3.80)	0.46	1.43 (0.23, 9.04)	0.71
Window	1		1	
Educational level				
Informal education	0.44(0.27, 0.74)	0.002*	1.22 (0.24, 6.24)	0.814
Low level	0.48 (0.28, 0.80	0.005*	0.86 (0.200, 3.73)	0.814
High level	1		1	
Occupation				
Government employer	1.46 (0.869, 2.44)	0.153	2.85(0.51, 16.02)	0.23
Private employer	0.81 (0.44, 1.41)	0.506	0.28(0.04, 1.91)	0.20
Homemaker	0.50 (0.29, 0.87)	0.014*	0.39 (0.09, 1.62)	0.19
Farmer	1		1	
Biomass use				
No	1.82(1.01, 3.21)	0.048*	6.04(1.59,22.87)	0.008*
yes	1		1	
Year of onset				
< 12 years	1.81 (1.19, 2.77)	0.006*	1.78 (0.96,4.77)	0.22
≥ 12 year	1		1	
Exacerbation -Menstruation				
Yes	2.05 (1.02, 4.12)	0.045	1.55 (0.55, 4.32)	0.405
No	1		1	
Relationship with HCP				
Poor	0.06 (0.02, 0.18)	0.000**	0.024(0.02, 0.23)	0.001*
Good	1		1	
Information by HCP				
No	0.48 (0.31, 0.73)	0.001*	1.28 (0.45, 3.60)	0.64
Yes	1		1	
Patient role of enablement				
Low	0.24 (0.16, 0.36)	0.000**	0.19 (0.08, 0.49)	0.001*
High	1		1	
Asthma severity				
Intermittent	1.22 (0.51, 2.95)	0.65	2.7 (0.21,35.2)	0.443
Mild persistent	1.57 (0.84, 2.91)	**0.158**	2.45 (0.27,22.1)	0.424
Moderate persistent	1.35 (0.76, 2.40)	0.299	2.2 (0.31,16.2)	0.429
Sever persistent ^R^	1		1	
Asthma medication				
ICS + SABA	2.45 (0.571,10.56)	**0.21**	1.19(0.09,16.2)	0.89
Oral +SABA	2.8 (0.55,14.23)	**0.20**	4.2 (0.18,95)	0.37
ICS + SABA+oral	1.9 (0.42,9.02)	0.39	6.3 (0.91,45)	0.95
Nonsteroid+SABA	1		1	
Concurrent medication Class				
CVD	0.79 (0.04,16.07)	0.884	2.3(0.42,12.5)	0.68
Endocrine drug	0.41(0.02, 8.30)	0.558	2.5 (0.47,13.7)	0.65
GI Drug	0.07 (0.002, 2.87)	**0.164**	0.17(0.00, 1,24)	0.49
Antipain	1.05 (0.05, 20.63	0.977	1.1 (0.45,26)	0.952
ART drugs	1		1	
Appropriateness of dose				
Optimal	3.10 (2.04, 4.71)	0.000**	2.16 (0.86, 5.44)	0.101
Sub-optimal	1		1	
HCP adherence to our guidelines				
Yes	4.87 (3.00, 7.89)	**0.001**	8.4(2.70, 26.01)	0.001**
No	1		1	
Appropriateness base on severity				
Yes	4.19 (2.75, 6.38)	0.00**	2.02 (0.72, 5.70)	0.18
No	1		1	
Comorbidity				
Yes	0.60 (0.42, 0.88)	0.008*	1.677 (0.487, 5.771)	0.41
No	1		1	
Adherence				
Low	0.304 (0.17, 0.52)	0.00**	0.16 (0.059, 0.481)	0.001**
High	1		1	

* *p* < 0.05,^**^ *p* **<** 0.001, ominous test,*p* < 0.001, VIF < 5, likelihood ratio = 158.445, log likelihood = − 126.091,*ICS* Inhaled corticosteroid, *CVD* Cardiovascular drug, *GI* Gasero intestinal, HCP Healthcare provider, *SABA* Salbutamol, *ART* Antiretroviral therapy

## Discussion

The long-term goal of asthma therapy is to reach and maintain well-controlled asthma. This can be achieved in a continuous cycle to measure, treatment adjustment, and evaluation of response. The purpose of continuous assessment is to diminish the burden on patients and their exacerbation risk, airway damage, and treatment-related side effects and to enhance patient QoL. In this multicenter public institutional-based study, the ACT measurement tool was applied to assess asthma control levels. This study’s findings reported that the overall mean (±SD) asthma control level was 16.5(±4).

The findings concerning the asthma control level showed that 28.9% of the participants had achieved the well asthma control levels. This finding was supported by studies conducted in Addis Ababa [32] and Saudi Arabia [33] reported that 32 and 31%, respectively, of the patients achieved the control levels. However, the number of patients in this study achieved much lower levels compared with the findings in Nigeria (37%) [21] and Cameroon (58%) [16]. The possible reasons for this might be the low number of respondents having the desired targets of well asthma control levels could be low medication adherence levels of the patients. Though the patients had regular ICS control medication prescriptions in their hand most of them used medications that they perceived to control the exacerbation symptoms immediately. However, the medication they used played no role in controlling the underlying inflammation of the airways. Moreover, the differences in guidelines, lack of patients’ knowledge about the disease and the presence of common comorbidities in patients with asthma importantly contribute to a lesser number of patients having controlled asthma conditions [20, 34]. In contrast, the results in this study showed that many sampled studies have achieved the required goals of the control status compared with the reports from Israel (7%) [35] and Italy (9.1%) [36]. This difference could be because the patients in this study had less comorbidity and were a higher prescription of anti-inflammatory drugs like ICS.

Being male has significantly increased the asthma control status and this is supported by a study conducted in America and Morocco [36, 37]. This difference could be attributed to sex hormones and males have no estrogen and menstruation-related asthma exacerbation. Moreover, the real practical situations appreciated in the study settings could also strengthen the differences in which females are more involved in asthma triggering conditions, activities, and work burdens than males. Married patients also had better asthma control levels and were consistent with the previous article [38]. This is because being married could reduce the conditions leading to stress. Stressful situations are among the risk factors for asthma exacerbation. It modulates and activates biological pathways involved in asthma pathophysiology and modulates inflammation through the release of hormones and neuropeptides that interact with immune cells [39]. Emotional events like illness or death of a loved one, marital issues, separation, divorce, and conflict have been linked to asthma and poorly controlled asthma [39, 40].

Using biomass fuel for food preparation was an important factor in reducing the capability to achieve the required levels of asthma control. The data found in the current study is quite consistent with the previous study [41], which stated that the use of black Carbone, which is a potential pollutant, increases the risk of getting exacerbation episodes and further imposes a negative impact on its control status. Pollutants could act on natural preventive respiratory much-clearance cells both directly and indirectly and induce the production of free oxygen radicals that potentially diffuse to the airway surfaces and cause inflammatory reactions. Besides, air pollutants could react with the atmospheric ozone (O3) layer that furtherly causing the production of Reactive Oxygen Species (ROS) and changes in the expression of claudins, the major components of tight junctions, thus leading to tight junction barrier permeability and Airway Hyper-responsiveness (AHR) [42, 43]. Another important factor, which possibly associated with the positive asthma control levels, was treating the patients as per the guidelines, and was comparable to the study conducted based on the GINA guidelines [44]. Therefore, this study implies that if clinicians adhere to the management guidelines and if patients could also adhere to the provided recommendations, the desired goal of asthma control levels would succeed.

The following variables were meaningfully associated with lower controlled asthma status; poor patients-provider relationship, patients with a low level of enablement role and poorly adhered to the ICS. Consequently, when the provider-patient relationships and communication have compromised the desired targets of treatment have not succeed well [45]. This implied that unless the relationship between the provider-patient is well built, the services provided to the clients would be potentially impaired because of lack of trust and dissatisfaction with the given services [46]. Likewise, low levels of enablement roles in the self-care practices of the patients also had unwanted outcomes of the asthma conditions and are linked to previous studies, which disclosed that the self-management training programs may improve asthma controls compared with patients on the usual care alone [46, 47]. A low level of enablement roles executed the patients could be the reason for further deteriorations in their trust in treatment and adherence. Meanwhile, once the patients’ perception of their treatment has changed, it will further compromise the outcomes because they thought that the interventions, they received played no role in their final treatment schedules. As a result, individuals with low roles in their care would have less frequent consult times and be unable to deal with the condition.

The patient’s poor adherence levels specifically to the ICS medication was found as a significant factor in their poor asthma control. This is further supported by findings from the UK that disclosed several patients were non-adherent and the majority stopped taking ICS once they felt better. So that more than half of the patients on ICS/LABA combination therapy were unable to achieve the targeted asthma control levels [48]. This low adherence may have been a result of unwanted steroid events, costs or unavailability of medications, inadequate patient health education, poor healthcare provider-patient relationship, and low level of patient satisfaction.

The study had several strengths. It was a real-life study that used well-validated tools to measure outcomes and it was a multicenter study, which increased the generalizability of the study. The study location, being a referral institution, was well capable in terms of resources. Furthermore, the study correlates subjective and objective findings. Additionally, a systematic random sampling technique was used. This could have reduced the source of bias in the study.

This study also has few limitations. Some responses in the questionnaires were patient subjective reports, which could have introduced social desirability bias either by under-reporting or exaggeration. Since ACT was administered by the operator patients might not want to freely admit the genuine extent of their symptoms. It is easier to conceal the truth from operator interview than self-administered and if a questionnaire is used in interview based, then the patient will be caught within the lie*.* Despite these limitations, our study was adequately powered and the findings compared well with those of other similar studies conducted worldwide.

## Conclusion

Our study revealed that a significant number of individuals presented with suboptimal asthma control. Concerning the potential independent predictors of asthma control level, male gender, who did not use biomass fuel for cooking, who are married, and patients whose HCP adherence to their guideline usage were significantly associated with higher odds of well-controlled asthma, whereas patients who have a poor relationship with health care providers, low level of the role of patient enablement and non-adherence to ICS were significantly associated with lower odd of well controlled. Therefore, healthcare providers should work to improve patients’ awareness of their medication’s adherence and avoid asthma triggering factors for decreasing the progression of the disease, and better level of asthma control.

## Data Availability

The datasets supporting the conclusions of this article are available upon request to the corresponding author. Due to data protection restrictions and participant confidentiality, we do not make participants data publicly available.

## Abbreviations

ACT: Asthma Control Test
AHR: Airway Hyper Responsiveness
AOR: Adjusted odds ratio
COPD: Chronic Obstructive Pulmonary Disease
CI: Confidence Interval
FHCSH: Felege Hiwot Comprehensive Specialized Hospital
GINA: Global Initiative for Asthma
HRQoL: Health-Related Quality of Life,ICS; Inhaled Corticosteroids,LABA; Long-Acting Beta-2 Agonists
PEI: Modified Patient Enablement Index
MARS-A: Medication Adherence Rating Scale for Asthma
QoL: Quality of Life
SABA: Short Acting Beta-2 Agonists,SD; Standard Deviation
TASH: TikureAnebesa Specialized Hospital
TGCSH: TibebeGhion Comprehensive
ROS: Reactive Oxygen Species,Specialized Hospital
UK: United Kingdom
UOGCSH: University of Gondar Comprehensive Specialized Hospital

## References

[1] Global initiative for asthma. Global Strategy for Asthma Management and Prevention. 2022. Available from: http://www.ginasthma.org.

[2] Reddel HK, B.L., Bateman ED, Brightling CE, Brusselle GG, Buhl R, et al. Global Initiative for Asthma (GINA) Strategy 2021–Executive summary and rationale for key changes. J Allergy Clin Immunol In Practice. 2022;10(1):1–18. 10.1016/j.jaip.2021.10.001 34718211

[3] See KC, Phua J, Lim TKJ (2016). Trigger factors in asthma and chronic obstructive pulmonary disease: a single-Centre cross-sectional survey. Singap Med J.

[4] Arruda LK (2001). Cockroach allergens and asthma. J Allergy Clin Immunol.

[5] Gidey K, A.A, Guizaw M, Franz KH, Potthoff A. Allergic sensitization to common antigens among Ethiopian asthmatic patients. Afr J Respir Med. 2015;10(2). Available from: https://www.africanjournalofrespiratorymedicine.com/articles/allergic-sensitization-to-common-antigens-among-ethiopian-asthmatic-patients.pdf.

[6] Platts-Mills TA (1997). Indoor allergens and asthma: report of the third international workshop. J Allergy Clin Immunol.

[7] Platts-Mills TA (1991). Epidemiology of the relationship between exposure to indoor allergens and asthma. Int Arch Allergy Immunol.

[8] Network., G.A (2018). The Global Asthma Report 2018.

[9] Boulet L-P, et al. The global initiative for asthma (GINA): 25 years later. Eur Respir J. 2019;54(2). Available from: https://erj.ersjournals.com/content/54/2/1900598.short. 10.1183/13993003.00598-2019 31273040

[10] Adeloye D (2013). An estimate of asthma prevalence in Africa: a systematic analysis. Croatian Med J.

[11] To T (2012). Global asthma prevalence in adults: findings from the cross-sectional world health survey. BMC Public Health.

[12] Tadesse DB (2020). Uncontrolled asthma in Ethiopia: a systematic review and meta-analysis. Adv Respir Med.

[13] Jia CE (2013). The asthma control test and asthma control questionnaire for assessing asthma control: systematic review and meta-analysis. J Allergy Clin Immunol.

[14] Gebremariam TH (2017). Level of asthma control and risk factors for poor asthma control among clinic patients seen at a referral Hospital in Addis Ababa, Ethiopia. BMC Res Notes.

[15] Rothe T (2018). Diagnosis and management of asthma–the Swiss guidelines. Respiration.

[16] Hugo MNB, et al. Assessment of asthma control using asthma control test in chest clinics in Cameroon: a cross-sectional study. Pan African Med J. 2016;23(1): Available from: https://www.ajol.info/index.php/pamj/article/view/138670. 10.11604/pamj.2016.23.70.8434 PMC4862776 27217894

[17] Busse WW (2012). Asthma outcomes workshop: overview. J Allergy Clin Immunol.

[18] Schatz M (2006). Asthma control test: reliability, validity, and responsiveness in patients not previously followed by asthma specialists. J Allergy Clin Immunol.

[19] Braido F (2016). Determinants and impact of suboptimal asthma control in Europe: the international cross-sectional and longitudinal assessment on asthma control (LIAISON) study. Respir Res.

[20] Braido F. Failure in asthma control: reasons and consequences. Hindawi. 2013;2013. Available from: https://www.hindawi.com/journals/scientifica/2013/549252/. 10.1155/2013/549252 PMC3881662 24455432

[21] Johbull J, Olaiya A, Efosa EJGJ (2012). Assessment of asthma control using asthma control test (ACT) and it relationship with lung function parameters. Med Sci.

[22] Kosse RC (2020). Asthma control and quality of life in adolescents: the role of illness perceptions, medication beliefs, and adherence. J Asthma.

[23] Thompson K, Kulkarni J, Sergejew AJ (2000). Reliability and validity of a new medication adherence rating scale (MARS) for the psychoses. Schizophr Res.

[24] Horne R, Weinman J, Hankins MJ (1999). The beliefs about medicines questionnaire: the development and evaluation of a new method for assessing the cognitive representation of medication. Psychol Health.

[25] Neame R, Hammond AJ (2005). Beliefs about medications: a questionnaire survey of people with rheumatoid arthritis. Rheumatology.

[26] Mahler C (2012). Patients' beliefs about medicines in a primary care setting in Germany. J Eval Clin Pract.

[27] Cohen JL (2009). Assessing the validity of self-reported medication adherence among inner-city asthmatic adults: the medication adherence report scale for asthma. Ann Allergy Asthma Immunol.

[28] Haughney J (2007). The use of a modification of the patient enablement instrument in asthma. Prim Care Respir J.

[29] Charlson M (1994). Validation of a combined comorbidity index. J Clin Epidemiol.

[30] Alzahrani YA, Becker EAJ (2016). Asthma control assessment tools. Respir Care.

[31] Tolvanen E, et al. Patient enablement after a single appointment with a GP: analysis of Finnish QUALICOPC data. J Prim Care Community Health. 2017;8(4):213–20. Available from: https://journals.sagepub.com/doi/full/10.1177/2150131917730211. 10.1177/2150131917730211 PMC5932738 28911251

[32] Zeru TG, Engidawork E, Berha ABJ. Assessment of asthma control and quality of life among asthmatic patients attending armed forces referral and teaching hospital, Addis Ababa, Ethiopia. Pulmonary Med. 2020;2020. 10.1155/2020/5389780 PMC7411494 32802503

[33] Al-Jahdali HH (2008). Asthma control assessment using asthma control test among patients attending 5 tertiary care hospitals in Saudi Arabia. Saudi Med J.

[34] Munoz-Cano R, et al. Follow-up of patients with uncontrolled asthma: clinical features of asthma patients according to the level of control achieved (the COAS study). Eur Respir J. 2017;49(3). Available from: https://erj.ersjournals.com/content/49/3/1501885.short. 10.1183/13993003.01885-2015 28254764

[35] Starobin D (2007). Asthma control and compliance in a cohort of adult asthmatics: first survey in Israel. Ramat Gan.

[36] Allegra L (2012). Real-life prospective study on asthma control in Italy: cross-sectional phase results. J Respir Med.

[37] Taylor D (2008). A new perspective on concepts of asthma severity and control. Eur Respir J.

[38] Ghanname I (2018). Factors associated with asthma control: MOSAR study (multicenter observational study of asthma in Rabat-Morocco). BMC Pulmon Med.

[39] Lietzén R (2011). Stressful life events and the onset of asthma. Eur Respir J.

[40] de Nijs SB, Venekamp LN, Bel EHJ (2013). Adult-onset asthma: is it really different?. Eur Respir Rev.

[41] Tiotiu AI (2020). Impact of air pollution on asthma outcomes. Int J Environ Res Public Health.

[42] Bontinck A, Maes T, Joos GJ (2020). Asthma and air pollution: recent insights in pathogenesis and clinical implications. Curr Opin Pulm Med.

[43] Kim BG (2018). Impact of ozone on claudins and tight junctions in the lungs. Environ Toxicol.

[44] Bateman E, Frith L, Braunstein GJ (2002). Achieving guideline-based asthma control: does the patient benefit?. Eur Respir J.

[45] Prip A (2018). The patient–healthcare professional relationship and communication in the oncology outpatient setting: a systematic review. Cancer Nurs.

[46] Thoonen BS, TRJ Van Den Boom G (2003). Self-management of asthma in general practice, asthma control and quality of life: a randomised controlled trial. Thorax.

[47] Buhl R, Price DJ (2004). Patients' perceptions of well-being using a guided self-management plan in asthma. Int J Clin Pract.

[48] Pavord ID (2017). The impact of poor asthma control among asthma patients treated with inhaled corticosteroids plus long-acting β2-agonists in the United Kingdom: a cross-sectional analysis. Prim Care Respir Med.

